# Oxidized OxyR Up-Regulates *ahpCF* Expression to Suppress Plating Defects of *oxyR-* and Catalase-Deficient Strains

**DOI:** 10.3389/fmicb.2019.00439

**Published:** 2019-03-07

**Authors:** Fen Wan, Jianhua Yin, Weining Sun, Haichun Gao

**Affiliations:** ^1^College of Laboratory Medicine, Hangzhou Medical College, Hangzhou, China; ^2^College of Biotechnology and Bioengineering, Zhejiang University of Technology, Hangzhou, China; ^3^Institute of Microbiology and College of Life Sciences, Zhejiang University, Hangzhou, China

**Keywords:** *Shewanella*, OxyR, oxidative stress, AhpCF, catalase

## Abstract

It is well established that in bacteria, such as *Escherichia coli*, OxyR is a transcriptional regulator that mediates the response to H_2_O_2_ by activating the OxyR regulon, which consists of many genes that play vital roles in oxidative stress resistance. In *Shewanella*, OxyR regulates, however, in both reduced and oxidized states, the production of H_2_O_2_ scavengers, including major catalase KatB and NADH peroxidase AhpCF. Here we showed that the *oxyR* mutant carried a plating defect manifested as division arresting, a phenotype that can be completely suppressed by an OxyR variant constitutively existing in oxidized form (OxyR^L197P^). This effect of OxyR^L197P^ could not be solely attributed to the increment in KatB production, since the suppression was also observed in the absence of KatB. Although expression of peroxidase CcpA was greatly activated by OxyR^L197P^, the contribution of the protein in alleviating plating defect was negligible. We eventually identified AhpCF as the critical factor, when produced at substantially elevated levels by OxyR^L197P^, to protect the cell from H_2_O_2_ attack. Our data indicate that AhpCF is a particularly important peroxidase in oxidative stress resistance in *Shewanella*, not only playing a compensatory role for catalase, but also by itself providing sufficient protection from killing of H_2_O_2_ generated abiotically.

## Introduction

Since the earth became an aerobic environment, one of the major challenges for living organisms has been the oxidative stress imposed by a variety of molecules that are produced by aerobic metabolism and by abiotic reaction. On the top of the list are reactive oxygen species (ROS), including superoxide (O_2_^-^), hydrogen peroxide (H_2_O_2_), and hydroxyl radical (OH), which cause damage to virtually all biomolecules such as DNA, RNA, lipid, and protein (Imlay, 2013). To adapt to or resist oxidative stress, both prokaryotes and eukaryotes have evolved many sophisticated antioxidant defense systems. In bacteria, cells employ enzymes like catalase, peroxidase, and superoxide dismutase to detoxify H_2_O_2_ and superoxide, respectively (Mishra and Imlay, 2012). Expression of the antioxidant defense systems is generally and concertedly controlled at the transcriptional level, a subject extensively studied in *Escherichia coli* (Imlay, 2015). OxyR, a transcriptional regulator of the LysR family, directly senses H_2_O_2_ via the oxidation of two conserved cysteine residues (Cys 199 and Cys 208 in *E. coli*) and the formation of an intramolecular disulfide bond (Zheng et al., 1998; Lee et al., 2004). In *E. coli*, oxidized OxyR activates the transcription of the antioxidant genes, including *katG* (hydrogen peroxidase I), *ahpCF* [Ahp, NADH peroxidase (originally named alkyl hydroperoxidase)], *ccpA* (cytochrome *c* peroxidase), *dps* (iron-sequestering protein), and *oxyS* (a small regulatory RNA) (Storz and Altuvia, 1994; Altuvia et al., 1997; Zheng et al., 2001; Khademian and Imlay, 2017). The Ahp system, consisting of AhpC and AhpF performing catalytic and AhpC-reactivating reactions, respectively, is the primary scavenger of microscale H_2_O_2_ (Seaver and Imlay, 2001), while KatG is a monofunctional catalase responsible for degrading a large amount of H_2_O_2_. AhpC contains two conserved cysteine residues to reduce H_2_O_2_ via the formation of a disulfide bond, which is subsequently recycled back by AhpF using NADH as reducing equivalent (Poole, 1996). CcpA, which is located in the periplasm, reduces H_2_O_2_ to water by using electrons from soluble cytochrome *c* (Atack and Kelly, 2007; Khademian and Imlay, 2017).

Although functioning as an activator, *E. coli* OxyR proteins in both oxidized and reduced forms possess DNA binding activity for a conserved binding motif comprising four regularly spaced ATAG elements (Toledano et al., 1994). OxyR is composed of a helix-turn-helix DNA binging domain (DBD) in the N-terminus and a regulatory domain in the C-terminus where lies the two conservative cysteine residues (Cys199 and Cys208). A C199S mutation locks OxyR in the reduced conformation and deprives of ability to activate *oxyS* (an untranslated regulatory RNA) transcription (Kullik et al., 1995; Zheng et al., 1998; Zhang et al., 2002).

*Shewanella*, a group of facultative Gram-negative anaerobes, inhabit in redox-stratified environments and are renowned for their remarkable respiratory abilities (Fredrickson et al., 2008). In recent years, members of the genus regarded as emerging pathogens for human and sea animals have been expanding (Janda and Abbott, 2014). Studies of *Shewanella*, mostly on genus representative *S. oneidensis*, suggest that the response to oxidative damage is quite different from that of *E. coli*. *S. oneidensis* is substantially more sensitive to H_2_O_2_ than *E. coli*, in concert with the high susceptibility to UV and ionizing radiation (Ghosal et al., 2005; Jiang et al., 2014; Li et al., 2014; Shi et al., 2015). In *S. oneidensis*, the primary catalase for H_2_O_2_ degradation is encoded by the *katB* gene; the KatB loss results in a plating defect on lysogeny broth (LB) plates (Jiang et al., 2014; Shi et al., 2015). This coincides with a similar scenario with an *E. coli* *oxyR* null mutant, whose plating defect is attributed to the lack of catalase induction (Christman et al., 1989). Unlike *E. coli* OxyR, the *S. oneidensis* counterpart functions as both a repressor and an activator for the *katB* gene (Jiang et al., 2014). As a result, compared to the wild-type, an *oxyR* null mutant produces KatB constitutively at levels between the conditions unchallenged and challenged by H_2_O_2_ (Shi et al., 2015; Wan et al., 2018). Despite this, the *S. oneidensis* *oxyR* mutant still carries a plating defect, which is even more severe than that of the *katB* mutant (Shi et al., 2015; Wan et al., 2017). Thus, there must be unknown factors responsible for the plating defect phenotype resulting from the OxyR loss in *S. oneidensis*.

In this report, we found that OxyR^L197P^, an OxyR variant that is locked into the oxidized state (Wan et al., 2018), and OxyR functioned, similarly, in alleviating plating defect of Δ*oxyR* but not of Δ*katB*. In Δ*oxyR*, OxyR^L197P^, the same as OxyR, activated expression of *katB* as a main means to scavenge H_2_O_2_, correcting the defect. In contrast, in Δ*katB* where AhpCF became an exclusive factor for H_2_O_2_ removal whereas contribution of other catalases and peroxidases was negligible, expression of *ahpCF* was elevated by OxyR^L197P^ but not OxyR to levels sufficiently high to compensate for the KatB loss. These findings provide new insights into the complementary roles of H_2_O_2_-scavenging enzymes in *S. oneidensis*.

## Materials and Methods

### Bacterial Strains, Plasmids and Culture Conditions

All bacterial strains and plasmids used in this study are listed in Table 1. *E. coli* and *S. oneidensis* were grown in LB (containing 1% tryptone, 0.5% yeast extract, and 0.5% NaCl) under the aerobic condition at 37 and 30°C for genetic manipulation. When necessary, following chemicals were added to the growth medium: 2,6-diaminopimelic acid (DAP), 0.3 mM; ampicillin, 50 μg/ml; kanamycin, 50 μg/ml; gentamycin, 15 μg/ml; and streptomycin, 100 μg/ml.

**Table 1:** Bacterial strains and plasmid used in this study.

Strain or plasmid	Description	Source or reference
*E. coli* strains		
DH5α	Host strain for cloning	Lab stock
WM3064	Δ*dapA*, donor strain for conjugation	W. Metcalf, UIUC
*S. oneidensis* strains	
MR-1	Wild type	ATCC 700550
HG1328	Δ*oxyR* derived from MR-1	Wan et al., 2017
HG1070	Δ*katB* derived from MR-1	Shi et al., 2015
HG0956-8	Δ*ahpCF* derived from MR-1	Shi et al., 2015
HG2178	Δ*ccpA* derived from MR-1	Shi et al., 2015
HG2750	Δ*tolR* derived from MR-1	Gao et al., 2017
HG0956-1070	Δ*katB*Δ*ahpC* derived from MR-1	Shi et al., 2015
HG1070-0725	Δ*katB*Δ*katG-1* derived from MR-1	This study
HG1070-4405	Δ*katB*Δ*katG-2* derived from MR-1	This study
HG1070-2178	Δ*katB*Δ*ccpA* derived from MR-1	This study
Plasmids		
pHGM01	Ap^r^Gm^r^Cm^r^,*att*-based suicide vector	Jin et al., 2013
pHG101	Km^r^, promoterless broad-host vector	Wu et al., 2011
pHGEI01	Km^r^, integrative *lacZ* reporter vector	Fu et al., 2013
pHGE-Ptac	Km^r^,IPTG-inducible expression vector	Luo et al., 2013
pHGE-Ptac-*ccpA*	Vector for inducible expression of CcpA	This study
pHGE-Ptac-*ahpCF*	Vector for inducible expression of *ahpCF*	This study
pHGEI01-P*katB*	Reporter vector carrying P*_katB_*-*lacZ*	This study
pHGEI01-P*ccpA*	Reporter vector carrying P*_ccpA_*-*lacZ*	This study
pHGEI01-P*dps*	Reporter vector carrying P*_dps_*-*lacZ*	This study
pHGEI01-P*ahpC*	Reporter vector carrying P*_ahpC_*-*lacZ*	This study
pHGEI01-P*katG-1*	Reporter vector carrying P*_katG-1_*-*lacZ*	This study


### Mutagenesis, Complementation of Mutant Strains

In-frame deletion strains for *S. oneidensis* were constructed according to the *att*-based Fusion PCR method as described previously (Jin et al., 2013). In brief, two fragments flanking the target gene were amplified by PCR with primers containing *attB* and the gene specific sequence, which were linked by a linker sequence via second round of PCR. The fusion fragments were integrated into plasmid pHGM01 by using Gateway BP clonase II enzyme mix (Invitrogen). The resultant plasmid was introduced in *E. coli* WM3064 and transferred to *S. oneidensis* by conjugation. Integration of the mutagenesis constructs into the chromosome was selected by resistance to gentamycin and confirmed by PCR. Verified trans-conjugants were grown in LB broth without NaCl and plated on LB supplemented with 10% sucrose. Gentamycin-sensitive and sucrose-resistant colonies were screened by PCR for deletion of the target gene. To facilitate growth of mutants, catalase (from bovine liver, Sigma) was added onto plates at the final resolution step for genes critical for survival through ROS. Mutants were verified by sequencing the mutated regions.

Genetic complementation of mutants with an apparent phenotype was performed with plasmids pHG101 or pHGE-Ptac as described before (Wu et al., 2011; Luo et al., 2013). For complementation of genes next to their promoter, a fragment containing the gene of interest and its native promoter was generated by PCR and cloned into pHG101. For the remaining genes, the gene of interest was generated by PCR and introduced into pHGE-Ptac under the control of promoter P*tac*. After sequencing verification, the resulting vectors were transferred into the relevant strains via conjugation.

### Microscopy

*Shewanella oneidensis* were cultivated to the mid-logarithmic (OD_600_ of 0.4) and spotted onto a glass slide containing LB medium. Motic BA310 light microscope (Motic, Xiamen, China) was employed to observe the cell morphology. Micrographs were captured with a Moticam 2306 charged-coupled-device camera.

### Spotting Assay

The spotting assay was used to evaluate the plating defect on LB plates. Cells of the log phase (OD_600_ of 0.4) were collected by centrifugation and adjusted to 10^9^ cell/ml, which was set as the undiluted (dilution factor 0). Ten-fold serial dilutions were prepared with fresh LB medium. Five microliters of each dilution was spotted onto LB plates. The plates were incubated for 24 h or longer in dark before being photographed. All experiments were repeated at least three times.

### Enzyme Activity Assay

Catalase and peroxidase activities were detected as described previously (Fridovich, 1984; Wayne and Diaz, 1986). For double staining to differentiate catalase and peroxidase, 2 ml of mid-log cultures were harvested by centrifugation and lysed by sonication. Forty microliters of total unboiled protein extract were loaded on a 10% non-denaturing polyacrylamide gel (PAGE) and run at 100 V at 4°C. After completion of the electrophoresis, the gel was washed in water and soaked in 100 ml of solution containing 0.01 ml of 30% H_2_O_2_, 50 mg of diaminobenzidine (Sigma) for 20 min. Then gel was washed by water and suspended in 5 mM H_2_O_2_, briefly washed in water and incubated in a solution containing 2% ferric chloride and 2% potassium ferricyanide. Catalase yielded clear bands on a green-stained background while peroxidase produced blue bands.

### β-Galactosidase Activity Assay

β-Galactosidase activity assay was used to determine gene expression. The sequence in sufficient length (∼400 bp) upstream of gene of interest was amplified and inserted in front of the full-length *E. coli lacZ* gene in plasmid pHGEI01 (Fu et al., 2014). The resulting vector was verified by sequencing and then transformed into *E. coli* WM3064 and then conjugated with relevant *S. oneidensis* strains. Cells at the mid-log phase were collected and centrifugation, washed with PBS, and treated with lysis buffer (0.25 M Tris/HCl, 0.5% Triton X-100, pH 7.5) for 30 min. Soluble protein was collected by centrifugation and applied for enzyme assay by adding o-nitrophenyl-β-D-galactopyranoside (ONPG) (4 mg/ml). β-Galactosidase activity was determined by monitoring color development at 420 nM with a Synergy 2 Pro200 Multi-Detection Microplate Reader (Tecan), presented as Miller units.

## Results

### *oxyR* Mutant Cells Die Quickly on LB Plates Without Morphological Changes

**FIGURE 1:**
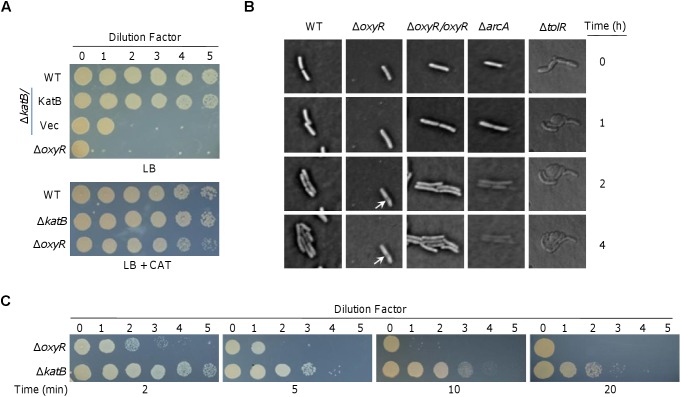
Characteristics of the plating defect of Δ*oxyR* and Δ*katB* on LB plates. **(A)** Spotting assay. Cells grown to the mid-log phase (OD_600_ of ∼0.4) were regarded as undiluted (dilution factor 0, ∼10^8^ CFU/ml) and were subjected to 10-fold series dilution. Five microliters of each dilution was dropped on LB plates. WT, the wild-type; Vec, empty vector. **(B)** Morphological observation under microscope. Cells of indicated strains at mid-log were spotted on slide with LB agar as described in the Materials and Methods. Pictures were captured at indicated times. The Δ*arcA* strain was incubated with 0.5 mM SDS. **(C)** Effects of catalase on rescuing viability of Δ*oxyR* and Δ*katB*. Catalase (2,000 U) was applied to the drops at the indicated times. Experiments were performed at least three times, and representative results were shown.

Deletion of the *S. oneidensis* *oxyR* gene (Δ*oxyR*) results in a serious plating defect (Jiang et al., 2014; Shi et al., 2015). Plating defect is a common phenotype that has been observed in many other bacteria, such as *E. coli*, whose OxyR proteins act as a positive regulator (Maciver and Hansen, 1996; Hahn et al., 2002). On LB plates, a drop (5 μl) of the culture containing 10^8^ CFU/ml grew as the wild-type, whereas none of a 10-fold dilution series showed visible growth (Figure 1A). In order to figure out the nature of this phenotype, we visualized growth of cells of relevant *S. oneidensis* strains on LB plates under a phase-contrast microscope. After spotted on LB agar, the wild-type cells were able to divide (Figure 1B), leading to colony formation on LB plates (Figure 1A). In contrast, Δ*oxyR* cells stayed in the single-cell state for 4 h and even longer, implicating that the mutation deprives cells of ability to proliferate (Figure 1B).

The elongated Δ*oxyR* cells became damaged in the cell envelope at the middle after 2 h incubation (Figure 1B). To investigate whether the plating defect is due to, at least in part, impaired cell envelope, we performed a comparative study with *arcA* and *tolR* mutants. In *S. oneidensis*, ArcA and TolR are a global regulator implicated in diverse processes and a protein involved in peptidoglycan recycling and cell division, respectively; the consequences caused by their loss are the severe defect in the outer-membrane and peptidoglycan layer (Gao et al., 2008, 2017). As a result, Δ*arcA* cells are very sensitive to SDS and Δ*tolR* cells form cell-chain with blebs (Gao et al., 2017; Wan et al., 2017). Under the microscope, we observed that Δ*arcA* cells retained ability to divide in the presence of 0.5% SDS (Figure 1B). However, the newly generated cells died quickly with the cell border increasingly blurring, and eventually becoming invisible. This phenomenon indicates that the cell envelope is dissolved by SDS, consistent with the defect in the outer-membrane. Expectedly, Δ*tolR* cells in chain quickly formed blebs at the cell surface without stopping division. The difference in the manners of death among Δ*oxyR*, Δ*arcA*, and Δ*tolR* cells, implies that the *oxyR* mutation may not affect cell envelope integrity.

To determine whether the plating defect phenotype is due to cell death, we performed catalase-rescuing assays with the Δ*oxyR* culture diluted 100-fold, which could not grow on LB plates (Figure 1A). Before and after culture dropping, catalase solution was applied to culture droplets at different times. As reported before (Shi et al., 2015), when catalase was added to LB plates before dropping, the plating defect of Δ*oxyR* could be fully corrected (Figure 1C). In the case of the application after culture dropping, however, the effects of catalase on the culture droplets were time-dependent (Figure 1C). When catalase was added 2 min later, growth was largely recovered. The rescuing effects became less effective and undetectable when catalase was applied 5 and 10 min after the culture dropping, respectively. These data indicate that the *S. oneidensis* Δ*oxyR* cells are nearly immediately damaged by H_2_O_2_ on LB plates, further supporting that cell envelope integrity and cell division are not the primary cause for death because defects in cell envelope do not kill cells rapidly and are not affected by cell density (Zerbib, 2017).

A similar but less severe plating defect has also been observed with the loss of KatB (Δ*katB*) (Shi et al., 2015) (Figure 1A). We therefore reasoned that catalase may rescue viability of the Δ*katB* strain in a similar manner. Indeed, Δ*katB* cells recovered ability to grow on LB plates if catalase was added in time (Figure 1C). Apparently, the rescuing effects of catalase on the Δ*katB* strain appear more effective than on the Δ*oxyR* strain. This is expected because the plating defect of the Δ*oxyR* strain is more severe. Nevertheless, it is clear that both strains die of the same damage caused by H_2_O_2_, which is generated abiotically on LB plates (Shi et al., 2015). These data collectively indicate that the plating defects of Δ*oxyR* and Δ*katB* strains are due to rapid cell death, rather than the inability to divide.

### KatB Is Not an Exclusive Factor for the Rescuing Effect of OxyR^L197P^ on the *oxyR* Mutant

**FIGURE 2:**
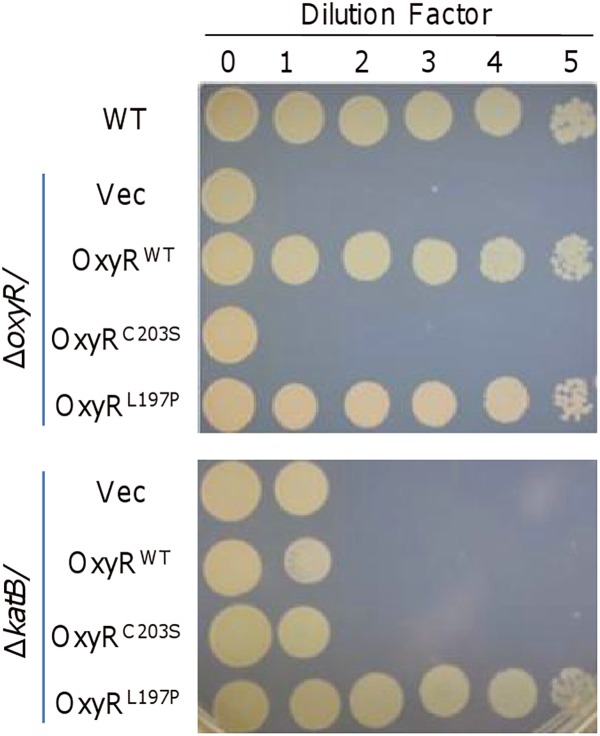
KatB is not an exclusive factor for the rescuing effect of OxyR^L197P^. The experiment was performed the same as described in Figure 1. Expression of OxyR^C203S^ and OxyR^L197P^ were driven by their own promoters in the background of Δ*oxyR* (upper) and Δ*katB* (lower). Experiments were performed at least three times, and representative results were shown.

To further investigate into mechanisms for the plating defect of the *oxyR* mutant, we compared the complementary effects of three OxyR variants, OxyR^WT^ (wild-type), OxyR^C203S^, and OxyR^L197P^ (Binnenkade et al., 2014; Jiang et al., 2014). In *S. oneidensis*, OxyR^WT^ proteins exist in both reduced and oxidized states, which could convert to each other through the formation of intramolecular disulfide bond between Cys-203 and Cys-212. While OxyR^C203S^ is locked in reduced form (as a repressor only for *katB* and *dps*) because of the inability to form disulfide bond, OxyR^L197P^ functions exclusively as an activator for all OxyR regulon members (Jiang et al., 2014; Wan et al., 2018). Plasmid pHG101 carrying the *oxyR* gene with respective mutations was used to produce OxyR^C203S^ and OxyR^L197P^, whose expression was driven by the *oxyR* own promoter (Wu et al., 2011). Clearly, both OxyR^WT^ and OxyR^L197P^ could completely eliminate the plating defect of Δ*oxyR* but OxyR^C203S^ failed (Figure 2).

Given that the plating defect is observed in strains lacking OxyR or KatB and overproduction of KatB, which can be achieved with OxyR^L197P^, could greatly improve the viability of the Δ*oxyR* strain (Wan et al., 2018), it is conceivable that KatB would be the key factor for survival of *S. oneidensis* on LB plates. To test this, we compared effects of OxyR^WT^, OxyR^C203S^, and OxyR^L197P^ on viability of the Δ*katB* strain (Figure 2). Expectedly, OxyR^C203S^ did not significantly alleviate the plating defect of Δ*katB*. A similar result was obtained with OxyR^WT^, supporting that the KatB loss is critically responsible for the plating defect. However, in the presence of OxyR^L197P^, Δ*katB* cells surprisingly displayed viability comparable to that of the wild-type (Figure 2). This phenomenon suggests that other unknown factors, which could be activated by OxyR^L197P^, are able to compensate for the KatB loss.

### CcpA Overproduced by OxyR^L197P^ Does Not Account for the Plating Defect

**FIGURE 3:**
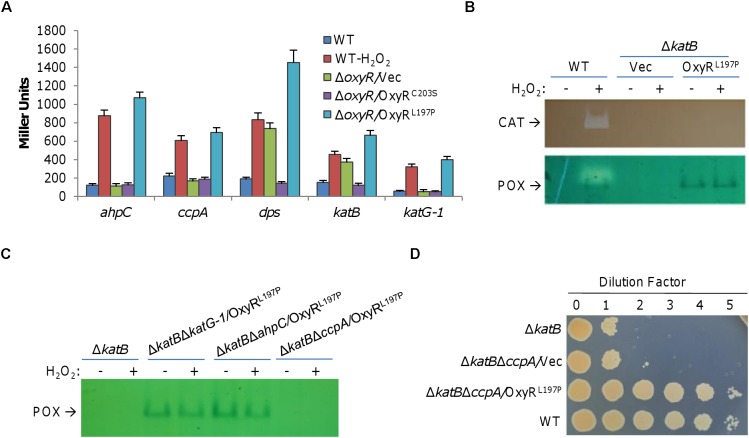
CcpA overproduced by OxyR^L197P^ does not account for the plating defect. **(A)** Expression of indicated genes in the presence of OxyR^C203S^ and OxyR^L197P^. Cells grown to the mid-log phase were treated with 0.2 mM H_2_O_2_ for 10 min or not, and then harvested for the assays. For Δ*oxyR* strains, H_2_O_2_ treatment did not affect expression and results from treated samples were shown. β-Galactosidase assays were carried out with *lacZ* reporters and activities were reported as the mean ± SD (*n* ≥ 4). **(B)** Double staining analysis. Cells of the mid-log phase were collected before and after 0.2 mM H_2_O_2_ treatment for 10 min. Proteins of indicated cells lysates were separated by native PAGE and stained for catalase (seen as clear zone) and peroxidase (seen as dark band) activities as indicated. **(C)** Peroxidase staining verified expression of peroxidase. Cells were collected before and after 0.2 mM H_2_O_2_ treatment for 10 min. Proteins of indicated cells lysates were separated by native PAGE and stained for peroxidase. **(D)** CcpA is not the main cause for plating defect alleviating. Droplet assay was used to verify the impact of CcpA on plating defect. For **B**, **C**, and **D**, experiments were performed at least three times, and representative results were shown.

The predicted *S. oneidensis* OxyR regulon is rather small comparing to those reported in other bacteria, comprising five members (Wan et al., 2018). In addition to *katB*, *ahpCF*, and *dps* mentioned above, two remaining members are *katG-1* (SO_0725) and *ccpA*, encoding catalase/peroxidase HPI and cytochrome *c* peroxidase, respectively. Single-gene mutants for all of these genes but *katB* are indistinguishable from the wild-type, with respect to growth in liquid LB and LB plates (Jiang et al., 2014; Shi et al., 2015). By using integrative *lacZ*-reporters (Jiang et al., 2014), we showed that *ahpC*, *ccpA*, and *katG-1* required OxyR^L197P^ for expression induction but were not affected by OxyR^C203S^ (Figure 3A). In contrast, expression of both *katB* and *dps* was affected negatively and positively by OxyR^C203S^ and OxyR^L197P^, respectively. Despite this difference, all predicted members of the OxyR regulon in the presence of OxyR^L197P^ were expressed at drastically increased levels, validating that OxyR^L197P^ functions as an activator only.

The causing agent of the plating defect is H_2_O_2_ that is generated abiotically on LB plates. As OxyR^L197P^ may up-regulate expression of genes for diverse functions, we focused on proteins that may functionally replace KatB. To this end, we performed a double staining for both catalase and peroxidase in the wild-type cells producing OxyR^L197P^ on native PAGE (Figure 3B). By this way, activity of catalase would be identified by a clear band, while peroxidase yields a blue band (Wayne and Diaz, 1986). Results revealed two bands: one for catalase, which is KatB as confirmed by the result with Δ*katB*/OxyR^L197P^, and the other for peroxidase (Figure 3B). To identify this peroxidase, we knocked out all candidate genes for peroxidases from the Δ*katB* background. As shown in Figure 3C, in the presence of OxyR^L197P^, the blue band remained in Δ*katB*Δ*katG-1* and Δ*katB*Δ*ahpC* but disappeared in Δ*katB*Δ*ccpA*, indicating that CcpA is the peroxidase. For validation, we placed the *ccpA* coding sequence under the control of isopropyl-β-D-thiogalactopyranoside (IPTG)-inducible promoter P*_tac_* within pHGE-Ptac and the expression vectors were introduced into the wild-type. Double staining revealed that the blue band became intensified with IPTG concentrations (Supplementary Figure S1). Thus, OxyR^L197P^ greatly enhances the production of CcpA, which was the only peroxidase that could be detected by the double staining method.

CcpA is a periplasmic cytochrome *c* peroxidase, catalyzing the reduction of H_2_O_2_, which is regarded as an alternative terminal electron acceptor in bacteria (Schütz et al., 2011; Khademian and Imlay, 2017). Because many important cellular components outside the *S. oneidensis* cytoplasm are vulnerable to H_2_O_2_ (Shi et al., 2015), CcpA may be critical for Δ*katB* survival. To test this, spotting assays of series dilution cultures prepared from relevant strains were performed (Figure 3D). Additional removal of CcpA (Δ*katB*Δ*ccpA*) did not further reduce viability of KatB-deficient cells on LB plates. More importantly, the Δ*katB*Δ*ccpA* strain with OxyR^L197P^, the same as Δ*katB*, recovered ability to grow on LB plates (Figure 3D). These results rule out the possibility that CcpA is the peroxidase that is able to compensate for the KatB loss.

### Ahp System Is Likely a Crucial Factor for Rescuing the Plating Defect

**FIGURE 4:**
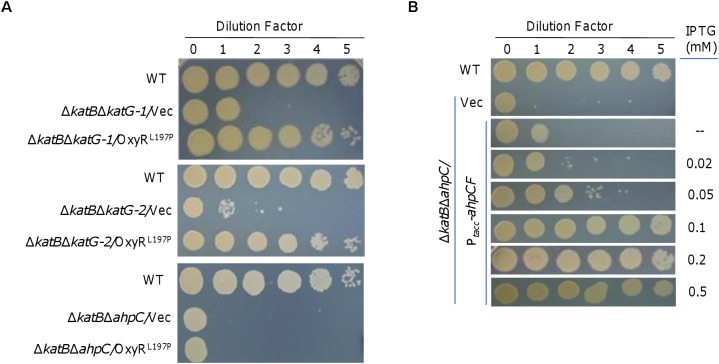
Ahp system is likely a crucial factor for rescuing the plating defect. **(A)** Spotting assay to screen for the crucial catalase and/or peroxidase that could correct the plating defect of Δ*katB*. Mid-log cultures of indicated strains were subject to 10-fold serial dilution, and 5 μl of each dilution was spotted onto plates. **(B)** Effects of AhpCF expressed to varying levels on the plating defect of Δ*katB*Δ*ahpC*. Expression of AhpCF was driven by IPTG-inducible P*_tac_*. In both **A** and **B**, experiments were performed at least three times, and representative results were shown.

Given that KatB and CcpA are the only catalase and peroxidase identified by the staining method on native PAGE, it is clear that the method is not sufficiently sensitive to detect all H_2_O_2_-degrading proteins encoded in the *S. oneidensis* genome. In *S. oneidensis*, peroxidase Ahp plays a protective role against oxidative stresses imposed by both H_2_O_2_ and organic peroxides (Li et al., 2014), whereas physiological significance of dual-function (catalase/peroxidase) HPI, KatG-1, remains unknown (Jiang et al., 2014). Consistently, we found that the Δ*katB*Δ*ahpC* strain was further impaired in viability on LB plates, whereas the Δ*katB*Δ*katG-1* strain displayed the plating defect the same as the Δ*katB* strain (Figure 4A). It was worth mentioning that KatG-2 (SO_4405), another dual-function HPI according to the genome annotation whose expression is OxyR-independent (Jiang et al., 2014; Wan et al., 2018), played a negligible role in influencing the plating defect (Figure 4A).

In order to identify the crucial catalase and/or peroxidase that could correct the plating defect of Δ*katB*, we expressed OxyR^L197P^ in the Δ*katB*Δ*ahpC*, Δ*katB*Δ*katG-1*, and Δ*katB*Δ*katG-2* strains and monitored viability on LB plates. The result revealed that OxyR^L197P^ fully recovered viability of the Δ*katB*Δ*katG-1* and Δ*katB*Δ*katG-2* strains, but had no noticeable effect on the Δ*katB*Δ*ahpC* strain (Figure 4A). These data suggest that Ahp is likely the peroxidase, when overproduced in the presence of OxyR^L197P^, that suppresses the plating defect of the Δ*katB* strain.

To confirm that Ahp in excess could compensate for the loss of KatB, we placed the *ahpCF* operon under the control of IPTG-inducible promoter P*_tac_* within pHGE-Ptac (Luo et al., 2013). It was immediately evident that the *ahpCF* operon expressed *in trans* had complementary effects on viability of the Δ*katB*Δ*ahpC* strain (Figure 4B). In the absence of IPTG, viability was nearly restored to that of the Δ*katB* strain because the promoter is slightly leaky (Luo et al., 2013; Shi et al., 2015; Meng et al., 2018). In the presence of IPTG less than 0.1 mM, viability of the Δ*katB*Δ*ahpC* strain was found to be improved increasingly with IPTG levels (Figure 4B). A full restoration was achieved with IPTG at 1 mM and above. These data thus validate that Ahp in excess rescues the growth defect resulting from compromised H_2_O_2_ degrading capacity.

## Discussion

The mechanism adopted by OxyR to cope with H_2_O_2_ stress is through activation of genes involved in adapting to and resisting oxidative stress, an understanding mostly built on the studies of the subject in *E. coli* (Imlay, 2013). In the absence of OxyR, expression of genes for major H_2_O_2_-degrading enzymes could not be activated, leading to plating defect, a well-documented common phenotype for *oxyR* mutants (Maciver and Hansen, 1996; Hahn et al., 2002). However, recent studies have revealed that OxyR proteins in some bacteria, including *S. oneidensis*, mediate expression of genes for major catalases and Ahp both positively and negatively (Loprasert et al., 2000; Tseng et al., 2003; Jiang et al., 2014; Wan et al., 2018). In this case, major H_2_O_2_-degrading enzymes are produced more in *oxyR* mutants than in the wild-type when cells are grown under normal conditions. Despite this, the OxyR loss still results in plating defect phenotype, at least in *S. oneidensis* (Jiang et al., 2014).

We have previously illustrated that the plating defect is due to H_2_O_2_ generated abiotically on LB plates (Shi et al., 2015). In this study, we uncovered that when properly diluted, cells lacking OxyR or major catalase KatB are unable to divide. Although membrane impairments are observed with extended incubation, they do not appear to be the cause for the plating defect. In *S. oneidensis*, KatB is the predominant force for H_2_O_2_ degradation, and its absence results in the plating defect. Given that effects of OxyR and KatB loss are highly similar, it is conclusive that the plating defect could be attributable to overall reduced H_2_O_2_-degrading capacity. In contrast, cells lacking Ahp, which is also a primary H_2_O_2_-degrading enzyme (Seaver and Imlay, 2001), are normal (Shi et al., 2015). Unlike catalase, Ahp decomposes multiple peroxides in addition to H_2_O_2_, including organic hydroperoxides (Niimura et al., 1995; Li et al., 2014). Despite this, it is clear that Ahp could not fully compensate for the loss of catalase in general, as shown here and in many other bacteria (Mongkolsuk et al., 2000; Seaver and Imlay, 2001; Cosgrove et al., 2007).

Given that the *oxyR* mutant produces KatB at levels higher than that in the wild-type grown under normal conditions, the catalase is not the exclusive factor for the plating defect (Shi et al., 2015). Indeed, we found here that Ahp is another critical factor for the plating defect. Clearly, enhanced production of Ahp, as a result of either OxyR^L197P^ up-regulation or manipulated over-expression, is able to correct the defect resulting from the KatB loss. The difference in rescuing effects of OxyR and OxyR^L197P^ on the plating defect of the *katB* mutant can be confidently explained by their redox states. OxyR proteins are always present in reduced and oxidized forms, which are in a dynamic equilibrium (Wan et al., 2018). When confronting H_2_O_2_ stress, OxyR_oxi_ outcompetes OxyR_red_ by higher affinity to target genes and activates expression. As OxyR^WT^ could not be completely oxidized and OxyR^L197P^ is locked in the oxidized state, the former is less effective in transcriptional activation as the latter.

Although the *S. oneidensis* genome encodes multiple H_2_O_2_-degrading enzymes, double staining for catalase and peroxidase has only identified activity of KatB and CcpA. Notably, activity for both enzymes is detected only in the wild-type cells challenged by H_2_O_2_ or with OxyR^L197P^. The failure to identify Ahp is likely a result of electron donors used in the analysis that could not serve as a cognate donor for Ahp (Trend et al., 2001). In parallel, activity of neither catalase nor peroxidase for HPI enzymes KatG-1 and KatG-2 is detected by the method, even in the presence of OxyR^L197P^. This may be readily explained by the lack of enzyme activity and extremely low expression, a scenario reported before (Jiang et al., 2014). Given that it is common that multiple enzymes for combating oxidative stress are encoded in bacteria and many of them remain functionally elusive (Mishra and Imlay, 2012), physiological impacts of KatG-1 and KatG-2 may still be worth investigation. For this, CcpA serves a good example. In *S. oneidensis*, CcpA is dispensable during aerobic growth since CcpA depletion leads to no phenotype (Jiang et al., 2014). However, the enzyme plays a protective role against oxidative stress under anaerobic conditions (Schütz et al., 2011). Similarly, CcpA is a potent degrader of H_2_O_2_ in anaerobic environment in *E. coli* (Khademian and Imlay, 2017). The mechanism underpinning this is that CcpA requires reductive activation for full activity, which depends on the absence of oxygen (Pulcu et al., 2012).

## Author Contributions

FW conducted and performed the experiments. WS was involved with the microscope result. FW and JY contributed to data discussion and analysis. FW designed and supervised the study, and wrote the manuscript with HG.

## Conflict of Interest Statement

The authors declare that the research was conducted in the absence of any commercial or financial relationships that could be construed as a potential conflict of interest.
