# Supplementary material for: Oxidized OxyR Up-Regulates ahpCF Expression to Suppress Plating Defects of oxyR- and Catalase-Deficient Strains
Source: Front Microbiol. 2019 Mar 7;10:439. doi: 10.3389/fmicb.2019.00439 (PMC6416212; doi:10.3389/fmicb.2019.00439)
Supplement: Supplementary file 1 [file Data_Sheet_1.docx]

***Supplementary information***

**Oxidized OxyR up-regulates *ahpCF* expression to suppress plating defects resulting from depletion of OxyR and catalase**


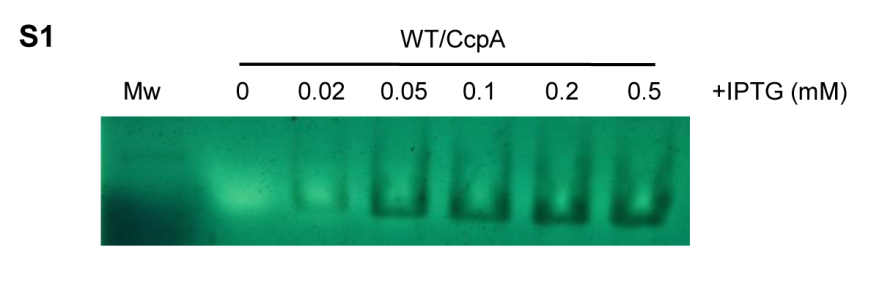


Figure S1. Visualization of CcpA activities by peroxidase gel staining. Cell extracts were loaded on 10% non-denaturing PAGE. IPTG of indicated concentration was used to induce CcpA expression. Mw represents protein standard marker.
